# DWI Intensity Values Predict FLAIR Lesions in Acute Ischemic Stroke

**DOI:** 10.1371/journal.pone.0092295

**Published:** 2014-03-21

**Authors:** Vince I. Madai, Ivana Galinovic, Ulrike Grittner, Olivier Zaro-Weber, Alice Schneider, Steve Z. Martin, Federico C. v. Samson-Himmelstjerna, Katharina L. Stengl, Matthias A. Mutke, Walter Moeller-Hartmann, Martin Ebinger, Jochen B. Fiebach, Jan Sobesky

**Affiliations:** 1 Center for Stroke Research Berlin (CSB), Charité-Universitätsmedizin, Berlin, Germany; 2 Max-Planck-Institute for Neurological Research, Cologne, Germany; 3 Fraunhofer MEVIS, Bremen, Germany; 4 Department of Radiology, Krankenhaus Ludmillenstift, Meppen, Germany; 5 Department of Neurology, Charité-Universtitätsmedizin, Berlin, Germany; 6 Department for Biostatistics and Clinical Epidemiology, Charité-Universitätsmedizin, Berlin, Germany; INSERM U894, Centre de Psychiatrie et Neurosciences, Hopital Sainte-Anne and Université Paris 5, France

## Abstract

**Background and Purpose:**

In acute stroke, the DWI-FLAIR mismatch allows for the allocation of patients to the thrombolysis window (<4.5 hours). FLAIR-lesions, however, may be challenging to assess. In comparison, DWI may be a useful bio-marker owing to high lesion contrast. We investigated the performance of a relative DWI signal intensity (rSI) threshold to predict the presence of FLAIR-lesions in acute stroke and analyzed its association with time-from-stroke-onset.

**Methods:**

In a retrospective, dual-center MR-imaging study we included patients with acute stroke and time-from-stroke-onset ≤12 hours (group A: n = 49, 1.5T; group B: n = 48, 3T). DW- and FLAIR-images were coregistered. The largest lesion extent in DWI defined the slice for further analysis. FLAIR-lesions were identified by 3 raters, delineated as regions-of-interest (ROIs) and copied on the DW-images. Circular ROIs were placed within the DWI-lesion and labeled according to the FLAIR-pattern (FLAIR+ or FLAIR−). ROI-values were normalized to the unaffected hemisphere. Adjusted and nonadjusted receiver-operating-characteristics (ROC) curve analysis on patient level was performed to analyze the ability of a DWI- and ADC-rSI threshold to predict the presence of FLAIR-lesions. Spearman correlation and adjusted linear regression analysis was performed to assess the relationship between DWI-intensity and time-from-stroke-onset.

**Results:**

DWI-rSI performed well in predicting lesions in FLAIR-imaging (mean area under the curve (AUC): group A: 0.84; group B: 0.85). An optimal mean DWI-rSI threshold was identified (A: 162%; B: 161%). ADC-maps performed worse (mean AUC: A: 0.58; B: 0.77). Adjusted regression models confirmed the superior performance of DWI-rSI. Correlation coefficents and linear regression showed a good association with time-from-stroke-onset for DWI-rSI, but not for ADC-rSI.

**Conclusion:**

An easily assessable DWI-rSI threshold identifies the presence of lesions in FLAIR-imaging with good accuracy and is associated with time-from-stroke-onset in acute stroke. This finding underlines the potential of a DWI-rSI threshold as a marker of lesion age.

## Introduction

In patients with acute ischemic stroke, the combination of a hyperintense lesion in diffusion weighted imaging (DWI) and the absence of a corresponding lesion in T2-weighted fluid-attenuated inversion recovery (FLAIR) imaging, the so called DWI-FLAIR mismatch, can predict the time from stroke onset <4.5 h [1]. This can be attributed to the time-dependent appearance of FLAIR-lesions within the first hours after stroke onset [2]–[4]. This finding is promising, as identification of patients eligible for thrombolysis with unknown stroke onset, e.g. in wake-up stroke, may be facilitated by specific imaging markers. However, the visual assessment of FLAIR lesions may be difficult [5], [6] and the automated analysis of FLAIR images is challenging owing to low contrast and partial volume effects [7]. Lesions on DW-images, on the other hand, show high contrast and can be easily delineated by automated software solutions [8] making DWI suitable for clinical stroke trials. In the present work, we hypothesized that DWI signal-intensity increases with the time-from-stroke-onset. To test this hypothesis we investigated, whether relative DWI intensity (DWI-rSI) is associated a) with the presence of hyperintensities in FLAIR-imaging and b) with time-from-stroke-onset.

## Materials and Methods

### Ethics Statement

All patients gave informed written consent prior to the study. The study was conducted according to the principles expressed in the Declaration of Helsinki and was approved by the authorized institutional review boards (IRB) of the University of Cologne and the Charité-Universitätsmedizin Berlin.

### Study Design

**Figure 1:**
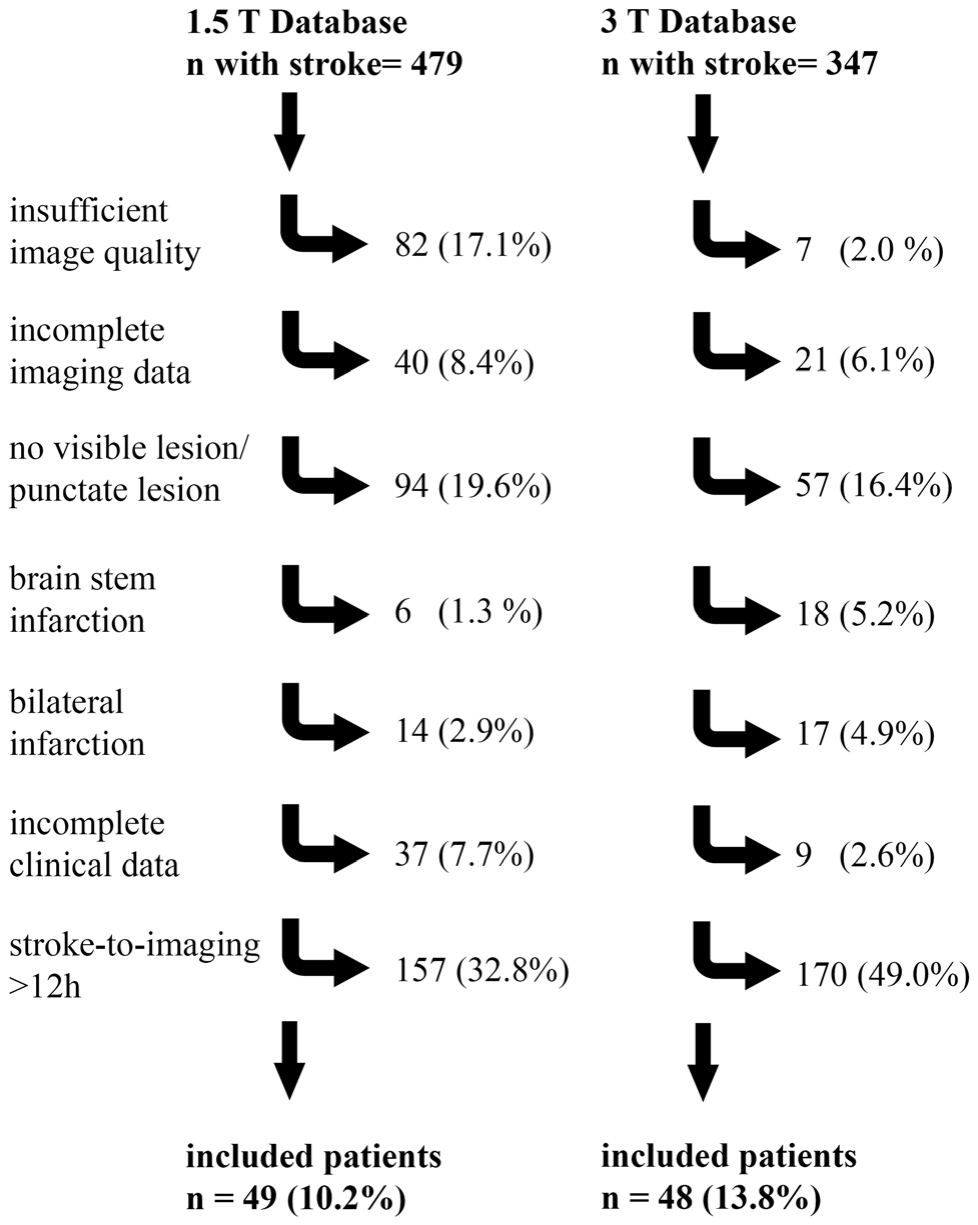
Database screening results and final study inclusion rate. In both databases the exclusion criterion with the highest exclusion rate was a stroke-to-imaging-time higher than 12 hours. In the 1.5 T database, the number of patients, which had to be excluded due to insufficient image quality (mainly of FLAIR images), was much higher (17.1%) than in the 3 T database (2.0%).

We performed a dual center retrospective observational imaging study. Imaging data including DW- and FLAIR-images were acquired from two stroke imaging databases: Group A, 1.5 T MR-imaging, University of Cologne, neurological imaging data base. Stroke patients available for the analysis were imaged consecutively between 2/2002 and 5/2004, in total 430 patients; Group B, 3 T MR-imaging, Charité-Universitätsmedizin Berlin, stroke imaging data base. Stroke patients available for the analysis were imaged consecutively between 3/2008 and 8/2010, in total 347 patients. Databases were screened and patients were included according to the following criteria: 1) clinically proven stroke, 2) confirmed symptom onset <12 h, 3) confirmed unilateral stroke lesion in DW-imaging, 4) available FLAIR imaging. Exclusion criteria were: 1) insufficient image quality, 2) incomplete clinical data, 3) punctate lesions and 4) brainstem infarctions. For a flow chart depicting the exclusions from the individual databases, see figure 1.

### Magnetic Resonance Imaging Hardware

MR-imaging was performed at 1.5 T on a Philips Gyroscan Intera Master whole-body system (Philips Medical Systems, Best, The Netherlands). At 3 T, a Magnetom Tim Trio whole-body system (Siemens Healthcare, Erlangen, Germany) was used.

### Magnetic Resonance Imaging Parameters

DW- and FLAIR imaging parameters were:

- At 1.5 T:
  1. DWI: single shot SE-EPI (TE: 96 ms, TR: 3560 ms, flip angle: 90°, matrix: 256×256, FoV: 230×230, b:0 and b:1000, pixel size: 0.9×0.9 mm^2^, slice thickness: 6 mm, interslice gap: 0.6 mm)
  2. T2-weighted FLAIR: (TE: 100 ms, TR: 6000 ms, TI: 2000 ms, flip angle: 90°, matrix: 256×256, FoV: 220×220, pixel size 0.9×0.9 mm^2^, slice thickness: 6 mm, interslice gap: 0.6 mm)
- At 3 T:
  1. DWI: single shot SE-EPI (TE: 93 ms, TR: 7600 ms, flip angle: 90°, matrix: 192×192, FoV: 230×230, b:0 and b:1000, pixel size 1.2×1.2 mm^2^, slice thickness: 2.5 mm)
  2. T2-weighted FLAIR: (TE: 100 ms, TR: 8000 ms, TI: 2370 ms, flip angle: 130°, matrix: 256×256, FoV: 220×220, pixel size 0.9×0.9 mm^2^, slice thickness: 5 mm, interslice gap: 0.5 mm).

### Data Postprocessing and Image Analysis

Co-registration and post-processing of DW- and FLAIR images was performed with VINCI, Version 2.63 (Max-Planck-Institute for Neurological Research, Cologne, Germany) [9]. DWI lesion volumina were assessed using MRIcron (Chris Rorden, http://www.mccauslandcenter.sc.edu/mricro/). At 3 T, DW images were resized in the z-axis to match FLAIR images.

**Figure 2:**
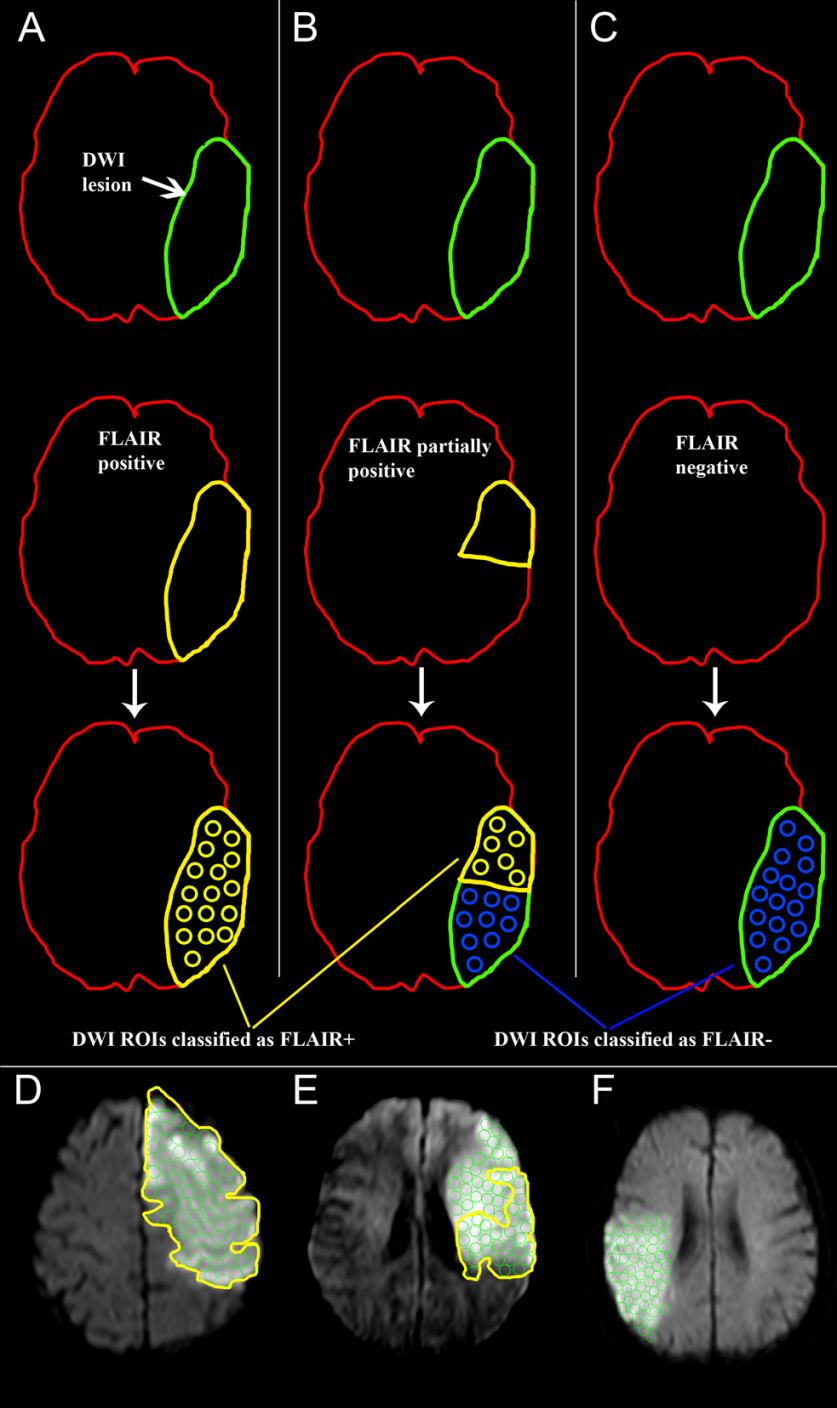
Labeling of lesions in DWI imaging according to the FLAIR pattern. A) In case of a FLAIR lesion encompassing the whole DWI-lesion, the hyperintensity was delineated as a region of interest (ROI) (A, second row). The FLAIR-ROI was then copied on the DWI and filled with 6 mm circular ROIs (A, third row)). These ROIs were classified as FLAIR+. B) In cases, in which the FLAIR-ROI did not completely match the DWI-lesion (B, second row), ROIs inside the FLAIR-ROI were classified as FLAIR+, and those outside as FLAIR- (B, third row). C) If no FLAIR lesion was identified (C, second row), the whole DWI lesion was filled with circular ROIs, which were classified as FLAIR- (C, third row). These steps were performed equally in ADC-maps. D), E) and F) show examples in analogy to the scheme, D) showing a patient, where the delineated FLAIR-ROI encompasses the whole DWI-lesion, E) depicting a patient, where the FLAIR-lesion only partially covers the DWI-lesion. Lastly, F) shows a patient, where all DWI-ROIs were labelled as FLAIR- in the absence of a visible FLAIR lesion.

In DWI, the slice with the largest lesion extent was identified visually and used for the complete further analysis. DW-images were co-registered to FLAIR images and the absence or presence of FLAIR lesions was assessed by three raters blinded to clinical data and DW-images (experience in stroke imaging is indicated for each rater; Rater 1, VIM: 3 years, Rater 2, ME: 5 years, Rater 3, JS: 10 years). Prior to the rating, raters were encouraged to look for subtle intensity changes by adjusting contrast and brightness of the images and to compare intensities of potentially hyperintense regions with the healthy contralateral hemisphere. Such subtle intensity changes were also rated as a FLAIR hyperintensity. The area of the FLAIR lesion was individually delineated by each rater and copied on the DW-images. Then, 6 mm regions of interest (ROIs) were placed within the whole DWI-lesion. Each ROI was labeled according to its position in regard to the FLAIR-ROI. If it was located inside the FLAIR lesion, it was labeled FLAIR+ (positive), if it was located outside of the FLAIR lesion, it was labeled as FLAIR- (negative). If a FLAIR lesion was absent, all DWI ROIs of this patient were labeled as FLAIR-. For a graphical overview of the analysis see figure 2. ROI-values were normalized as a ratio: [100% x (mean ROI value/mean value of the unaffected hemisphere)], taken from a slice at the height of the lateral ventricles and above the putamen encompassing the corona radiata. In cases of cerebellar infarction, a ROI of the contralateral cerebellar hemisphere was used to normalize the ROI values. Above steps were performed equally for apparent diffusion coefficient (ADC)-maps.

### Statistical Analysis

Owing to skewed distribution of some variables, results are presented as median and interquartile range (IQR) if not indicated otherwise. Differences in clinical data between groups were assessed using the Mann-Whitney U rank sum test.

Agreement between raters for the identification of FLAIR-hyperintensities was analyzed using free-marginal kappa [10], [11]. Kappa values were evaluated as suggested by Landis and Koch [12].

ROI-analysis was performed on patient level and separately for the two centres. We used mean ROI-values per patient and per rater for positive ROIs and negative ROIs separately:

a) If all raters had some ROIs of a patient classified as having a positive FLAIR status, we used only the mean of the positive values and classified the patient as having a positive flair status.

b) If not all raters found positive ROIs for a patient, but all raters had ROIs classified as negative, we used only the mean of the negative ROI-values and classified the patient as negative. *

c) Only for some patients one of the raters classified all ROIs in another category as the other raters. For them we used the classification of the two corresponding raters and set the ROI value for the rater not corresponding to missing.

In the next step, the ability of a relative DWI-intensity threshold to predict the presence of corresponding FLAIR-hyperintensities was analyzed using an unadjusted receiver operating characteristics (ROC) curve analysis. The area under the curve (AUC) and the 95% confidence limits for the raters are reported. To get the optimal threshold, the Youden Index was used ( [13]. Sensitivity, specificity, and predictive values for the optimal thresholds are also reported.

To adjust for possible confounders, a multiple logistic regression model with the dependent variable “FLAIR-status” and independent variables “lesion volume”, “sex”, “thrombolysis” and “NIHSS” was used as a basic model (m0). In the additional model 1 (m1), “age” was added. Finally, different models were compared with regard to their ability to discriminate individuals in their FLAIR-status:

i) In model 2 (m2), we added “time-from-stroke-onset” to the m1 model.

ii) In model 3 (m3), we added the ROI-intensity for each rater separately to the m1 model. Paired sample statistical techniques were used for the comparison of two models. The method exploits the mathematical equivalence of the AUC to the Mann-Whitney U-statistic [14]. The ROC curves were calculated using SPSS Statistics 21, Release Version 21.0.0.0 (SPSS, Inc., 2012, Chicago, IL, www.spss.com). The comparisons of ROC curves and the linear mixed models were done using SAS software, Version 9.3 of the SAS System for Windows. (2010 SAS Institute Inc., Cary, NC, USA).

For analyzing the association between DWI-rSI and time-from-stroke-onset we calculated the mean DWI intensity over all ROIs and raters for every patient and used unadjusted and adjusted correlation analysis (Spearman's rank correlation) and a multiple linear regression analysis adjusted for “age”, “lesion volume” and “thrombolysis”. We calculated multiple linear regressions with (log-transformed) “mean DWI-value” as dependent variable and “age”, “thrombolysis” and (log-transformed) “lesion volume” as independent variables. Mean DWI intensities and lesion volume values were log-transformed to overcome the skewness in the distribution of the values. We analyzed the adjusted association between mean DWI-rSI and time-from-stroke-onset by analyzing the association of the residuals from the regression analysis with time-from-stroke-onset.

Above steps were performed equally for ADC-maps.

## Results

In group A (1.5 T), 49 patients (16 females) and in group B (3 T) 48 patients (22 females) were included in the analysis. Median values for clinical data were (Group A/Group B): Time-from-stroke-onset (h) was 2.4/2.0; age (years) was 62/74; NIHSS (points) was 8/5 and the lesion volume (ml) was 22.9/6.8. The two groups differed significantly in age, NIHSS and lesion volume, but not in the time-from-stroke-onset. Five patients had cerebellar infarction (1 in group A and 4 in group B). Detailed patient data are shown in Table 1.

**Table 1:** Clinical data, imaging data and comparison of patient groups.

	Group A (1.5 T)	Group B (3 T)	p
Patients (n)	49	48	
Time Stroke to Imaging (h)	2.4 (1.7–5.3)	2.0 (1.0–3.7)	0.115
Age (y)	62 (52–67)	74 (64–84)	<0.001*
NIHSS (points)	8 (6–13)	5 (4–13)	0.047*
Stroke lesion volume (mm3)	22.9 (9.2–45.1)	6.8 (2.5–21.4)	<0.001*
*Imaging time window after stroke*			
0–4.5 h	34 (69.4%)	39 (81.3%)	
4.6–6 h	4 (8.2%)	3 (6.3%)	
6.1–12 h	11 (22.4%)	6 (12.5%)	
Thrombolysis rate	25 (51%)	26 (54.2%)	
*Acute visible vessel occlusion^a^*	21 (42.9%)	27 (56.3%)	
ACA	0	5	
MCA	13	22	
PCA	1	1	
ICA/CCA	7	1	
VA/BA	1	2	
*Lesion location*			
ACA-territory	1 (2.0%)	1 (2.1%)	
MCA-territory	45 (91.8%)	39 (81.3%)	
PCA-territory	2 (4.1%)	4 (8.3%)	
Cerebellum	1 (2.0%)	4 (8.3%)	

Data are given as median and IQR (interquartile range); Groups were compared using the Mann-Whitney U rank sum test, significant differences are marked by an asterisk; n, number; h, hours; y, years; ACA: anterior cerebral artery; MCA: middle cerebral artery; PCA: posterior cerebral artery; ICA/CCA: internal/common carotid artery; VA: vertebral artery; BA: basilar artery. ^a^ =  if patients had occlusion in two different vessels at the same time (e.g. ICA and MCA), occlusion was indicated for both vessels.

Interrater agreement for the rating of FLAIR images as positive or negative for hyperintensities was substantial for both group A and group B with a kappa value of 0.62/0.69 (overall agreement was 81%/85%).

In the unadjusted ROC curve analysis, relative DWI-intensities performed well in discriminating hyperintensities in FLAIR imaging in both groups and for all 3 raters (results for groups and 3 raters; Group A: AUC 0.84, 0.91, 0.76 [mean: 0.84]; Group B: AUC 0.87, 0.86, 0.83 [mean: 0.85]). The Youden-Index identified comparable optimal relative DWI-intensity thresholds for both groups for all 3 raters (in %; Group A: 162, 158, 167, mean: 162; Group B: 163, 161, 159, mean: 161). In contrast, relative ADC-intensity values performed worse (group A: AUC 0.56, 0.55, 0.64; group B: AUC 0.74, 0.80, 0.77). Detailed data including sensitivity, specificity, positive and negative predictive value for the identified thresholds are listed in Table 2.

**Table 2:** Detailed results of the unadjusted ROC analysis for all 3 raters at 1.5 and 3 T.

	AUC (95%CI)	Threshold (%)	Sensitivity (%)	Specificity (%)	PPV (%)	NPV (%)
1.5 T DWI						
Rater 1 (n = 49)	0.84(0.72–0.95)	162	63.0	95.5	94.4	67.7
Rater 2 (n = 47)	0.91(0.84–0.99)	158	80.0	86.4	87.0	79.2
Rater 3 (n = 49)	0.76(0.63–0.89)	167	44.4	100.0	100.0	59.5
mean	0.84	162	62.5	93.7	93.8	68.8
3 T DWI						
Rater 1 (n = 48)	0.87 (0.77–0.89)	163	80.0	82.6	83.3	79.2
Rater 2 (n = 47)	0.86 (0.76–0.97)	161	79.2	78.3	79.2	78.3
Rater 3 (n = 46)	0.83 (0.71–0.95)	159	78.3	78.3	78.3	78.3
mean	0.85	161	79.2	79.7	80.3	78.6
1.5 T ADC						
Rater 1 (n = 49)	0.56 (0.40–0.73)	64	70.4	45.5	61.3	55.6
Rater 2 (n = 48)	0.55 (0.38–0.71)	69	53.8	68.2	66.7	55.6
Rater 3 (n = 49)	0.64 (0.49–0.80)	78	44.4	90.9	85.7	57.1
mean	0.58	70	56,2	68,2	71,2	56,1
3 T ADC						
Rater 1 (n = 48)	0.74 (0.59–0.88)	65	48.0	100.0	100.0	63.9
Rater 2 (n = 47)	0.80 (0.67–0.94)	60	70.8	87.0	85.0	74.1
Rater 3 (n = 46)	0.77 (0.62–0.91)	61	69.6	87.0	84.2	74.1
mean	0.77	62	62,8	91,3	89,7	70,7

At both 1.5 and 3 T, the ability of a relative DWI- or ADC threshold to predict the presence of lesion in FLAIR-imaging was investigated individually for 3 raters. The given threshold is the optimal relative intensity value cutoff determined by the Youden-Index. For each threshold, the corresponding sensitivity, specificity, PPV and NPV and their means are shown. 95% confidence intervals are given for each individual AUC. DWI-rSI performed better in discriminating hyperintensities in FLAIR imaging than ADC-rSI. AUC, Area under the curve; PPV, positive predictive value; NPV, negative predictive value.

**Figure 3:**
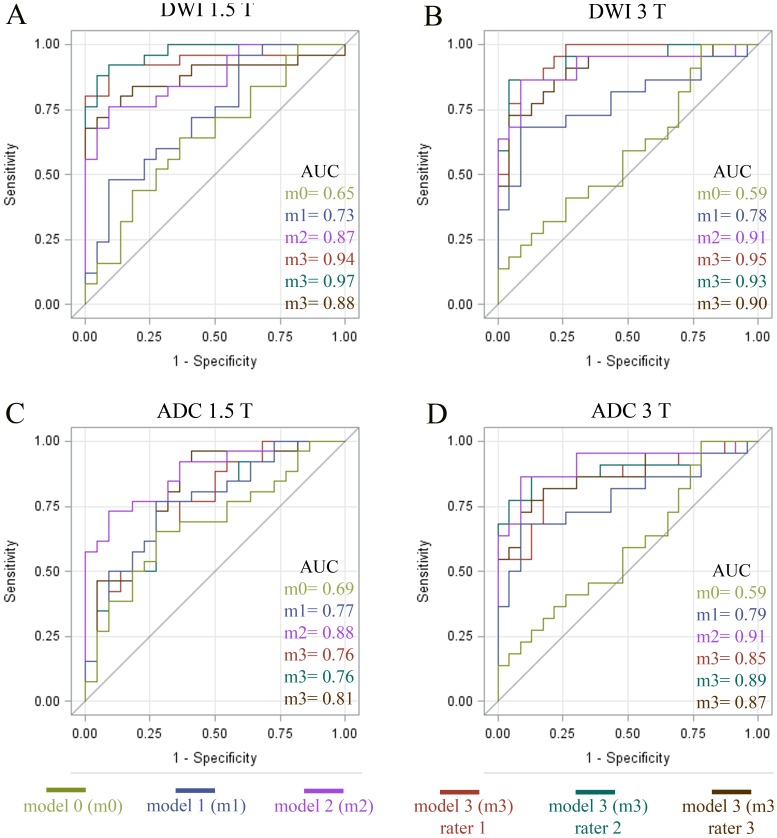
Adjusted ROC curves for the detection of presence of FLAIR-lesions by a relative DWI- and ADC-threshold. ROC-curves belonging to the detailed data presented in table 3 and 4 (please see legends of table 3 and 4 for further details). DWI-models for Group A (1.5 T) and B (3 T) (A,B) and ADC-models for Group A and B (C,D).

In the adjusted ROC-analysis for DWI, the basic model m0 including “lesion volume”, “sex”, “thrombolysis” and “NIHSS” had only a weak discrimination value for FLAIR status (AUC; Group A = 0.65; Group B = 0.59). The m1 model, where information on “age” was added, had a higher discrimination value (AUC; Group A = 0.73; Group B = 0.78). Adding “time-from-stroke-onset” to model m1 led to a further (significant) increase of the discrimination (model 2; AUC; Group A = 0.87; Group B = 0.91). Adding the ROI values for each rater as a variable in the m1-model, the increase was also significant in comparison with the m1 model and the discrimination value was comparable to the model 2 (model 3; mean AUC; Group A: 0.93; Group B: 0.93). Detailed data including p-values for the model comparison are listed in table 3. ROC-curves for model m0, m1, m2 and m3 for each rater are shown in figure 3.

**Table 3:** Detailed results of the adjusted ROC analysis for DWI and all 3 raters at 1.5 and 3 T.

	AUC(95% CI)	P for comparison with m1	P for comparison with m2
**1.5 T (n = 47)**			
Model 0 (m0)^a^	0.65 (0.49–0.81)	0.245	
Model 1 (m1)^b^	0.73 (0.59–0.88)		
Model 2 (m2)^c^	0.87 (0.78–0.97)	0.033	
Rater 1 model 3 (m3)^d^	0.94 (0.87–1.00)	0.004	0.075
Rater 2 m3^d^	0.97 (0.93–1.00)	<0.001	0.020
Rater 3 m3^d^	0.88 (0.77–0.98)	0.040	0.760
**3 T (n = 45)**			
m0^a^	0.59 (0.42–0.76)	0.017	
m1^b^	0.78 (0.64–0.93)		
m2^c^	0.91 (0.78–0.97)	0.031	
Rater 1 m3^d^	0.95 (0.89–1.00)	0.014	0.343
Rater 2 m3^d^	0.93 (0.86–1.00)	0.019	0.369
Rater 3 m3^d^	0.90 (0.80–0.99)	0.052	0.619

a m0: adjusted model, adjusted for lesion volume, sex, thrombolysis, NIHSS).

b m1: m0 additionally adjusted for age.

c m2: m1 additionally adjusted for time (stroke-to-imaging).

d m3: m1 and rater specific DWI-ROI values.

At both 1.5 and 3 T, adding the ROI-values for each rater (model 3[m3]) as a variable led to good accuracy for the prediction of FLAIR-hyperintensities for each rater in comparison with the basic models (m0 and m1). The AUC was comparable to m2, which was based on “time-from-stroke-onset”. Lack of a significant difference between m2 and m3 emphasizes the close association between time-from-stroke-onset and relative DWI-values. Please see figure 3 for the respective ROC-curves for each model. AUC, Area under the curve; ROI, Region of Interest.

For ADC in contrast, adding of ROI values in the m3 model did not increase the discrimination value. On the contrary, model 3 performed even worse than model 2, which was based on “time-from-stroke-onset”. Detailed data including p-values for the model comparison are listed in table 4. ROC-curves for model m0, m1, m2 and m3 for each rater are shown in figure 3.

**Table 4:** Detailed results of the adjusted ROC analysis for ADC and all 3 raters at 1.5 and 3 T.

	AUC(95% CI)	P for comparison with m1	P for comparison with m2
**1.5 T (n = 48)**			
Model 0 (m0)^a^	0.69 (0.53–0.84)	0.257	
Model 1 (m1)^b^	0.77 (0.63–0.90)		
Model 2 (m2)^c^	0.88 (0.79–0.97)	0.033	
Rater 1 model 3 (m3)^d^	0.76 (0.62–0.90)	0.720	0.036
Rater 2 m3^d^	0.76 (0.63–0.90)	0.817	0.039
Rater 3 m3^d^	0.81 (0.69–0.94)	0.340	0.272
**3 T (n = 45)**			
m0^a^	0.59 (0.42–0.76)	0.017	
m1^b^	0.79 (0.65–0.93)		
m2^c^	0.91 (0.82–1.00)	0.031	
Rater 1 m3^d^	0.85 (0.74–0.97)	0.249	0.374
Rater 2 m3^d^	0.89 (0.78–1.00)	0.111	0.734
Rater 3 m3^d^	0.87 (0.75–0.98)	0.130	0.455

a m0: adjusted model, adjusted for lesion volume, sex, thrombolysis, NIHSS).

b m1: m0 additionally adjusted for age.

c m2: m1 additionally adjusted for time (stroke-to-imaging).

d m3: m1 and rater specific ADC-ROI values.

In contrast to DWI (see table 3), adding the ADC-ROI-values for each rater (model 3[m3]) as a variable led to only a bad to moderate accuracy for the prediction of FLAIR-hyperintensities for each rater in comparison with the basic models (m0 and m1). The AUC was even inferior to m2, which was based on “time-from-stroke-onset”. Thus, ADC maps cannot reliably predict FLAIR-hyperintensities in contrast to DWI-maps. Please see figure 3 for the respective ROC-curves for each model. AUC, Area under the curve.

**Figure 4:**
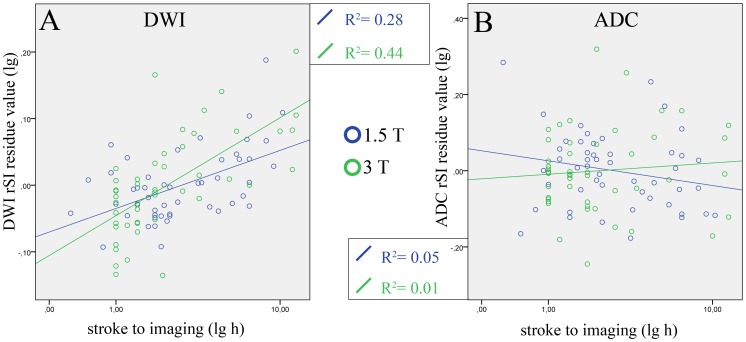
Adjusted linear regression analysis to evaluate the association of relative DWI-intensity and time-from-stroke-onset. Adjusted linear regression analysis was performed to identify a possible association of relative (A) DWI-intensity and (B) ADC-intensity (y-axis,) and time-from-stroke-onset (x-axis) at 1.5 T (blue circles) and 3 T (green circle). At both field strengths, a significant association was found for DWI (A) with moderate adjusted Rsquare values (1.5 T: 0.28; 3 T: 0.44). Adjusted correlation (Spearman's rank correlation) was: 1.5 T = 0.45 (p<0.001), 3 T = 0.69 (p<0.001). In contrast, no association was found for ADC-maps (B) with adjusted Rsquare values near zero (1.5 T: 0.04; 3 T: 0.01) and weak to no adjusted correlation (1.5 T = −0.22, 3 T = 0.05). Plots are shown in logarithmic scale.

In the unadjusted correlation analysis, a significant moderate to good correlation between mean relative DWI intensity and time-from-stroke-onset was found (Group A: r_s_ = 0.54 (p<0.001); Group B: r_s_ = 0.73 (p<0.011). The adjusted correlation confirmed a moderate to good correlation (Group A: r_s_ = 0.45 (p<0.001;, Group B: r_s_ = 0.69 (p<0.011) with a significant moderate fit in the linear regression analysis (see figure 4).

For ADC in contrast, no correlation was found between ADC and time-frome-stroke-onset (unadjusted analysis: Group A r_s_ = −0.25, Group B r_s_ = 0.07; adjusted analysis: Group A r_s_ = −0.22, Group B r_s_ = 0.05) and no fit was present in the linear regression analysis (see figure 4).

## Discussion

We report on the ability of a relative DWI-intensity threshold to discriminate with good accuracy between absence or presence of hyperintensities in corresponding FLAIR-images at both 1.5 and 3 T. The presence of FLAIR-hyperintensities was determined by 3 readers, who showed substantial interrater agreement. At both field strengths, similar DWI-intensity thresholds were identified. In addition, DWI intensity showed a significant association with time-from-stroke-onset.

To date, patients with unknown time from stroke onset are excluded from intravenous thrombolysis [15], [16]. As stroke incidence rates are higher in the morning hours compared to the rest of the day [17], patients *in theory* eligible for thrombolysis are *in practice* excluded from thrombolysis if time of stroke onset is unknown. Strategies to identify patients eligible for thrombolysis by MRI have been a major focus of interest in stroke research [18]. While ischemic lesions are visible in DWI as early as several minutes after stroke [19], lesions in T2-weighted FLAIR imaging show a later appearance, where the majority of patients displays FLAIR lesions only after several hours of stroke [2] [3] [4]. Thus, it is not surprising that the DWI-FLAIR mismatch allows for the allocation of patients to the current thrombolysis time window (i.e. <4.5 h after stroke) with a high specificity and a high positive predictive value [1]. The use of the DWI-FLAIR mismatch, however, mainly relies on the visual assessment of FLAIR hyperintensities. On one hand, visual qualitative assessment of FLAIR-images is challenging [6]. On the other hand, an automated analysis and the delineation of lesions in FLAIR imaging may be difficult owing to low contrast and partial volume effects [7]. This explains, why the use of quantitative relative FLAIR-intensity values has led to heterogenous results [3] [4] [20] [21]. We therefore hypothesized that DWI-maps might yield a surrogate of FLAIR imaging and we were able to show that a DWI based threshold predicts the presence of a FLAIR lesion with good performance across two different MR field strengths and three independent raters. In addition, mean DWI intensity of the lesion showed significant association with time-from-stroke-onset. These results strongly indicate two important points: *First*, DWI-intensity might exhibit a time-dependent increase after onset of ischemia in the acute phase. This specifies previous findings by Petkova et al. that described different DWI intensity values in patient samples stratified according to time-from-stroke-onset [4]. In that work, DWI-rSI was able to allocate patients to the thrombolysis time window <3 h (AUC: 0.75), but did not perform better than ADC-rSI (AUC: 0.74). It should be noted, however, that the authors projected the DWI lesion as a mask on ADC-maps and did not delineate the lesion in ADC-maps individually to derive rSI values. The reported threshold for DWI for the allocation of patients to the thrombolysis window was 19% using the formula “(DWI-lesion - contralateral value)/(DWI-lesion + contralateral value) × 100”. Recalculating our threshold of 160% rSI according to that formula leads to a similar threshold of 23% corroborating the pivotal association between FLAIR-appearance, DWI-rSI and time-from-stroke-onset.

*Second*, our results indicate that DWI-intensity is associated with tissue fate. The finding that a DWI-intensity threshold allows the prediction of FLAIR hyperintensity suggests that DWI-intensity follows a pathophysiologically driven time course similar to the manifestation of FLAIR lesions rather than simply increasing linearly with time. This is corroborated by previous results showing that a certain DWI-intensity threshold (118%) was able to predict permanent infarction as shown in an acute stroke sample imaged by MRI and comparative positron emission tomography [22]. This is important, as the DWI/FLAIR mismatch is limited by a high percentage of patients showing FLAIR positive lesion early after stroke [1]. Recently, it has been reported that this is even more pronounced at 3T, where a high percentage of patients showed FLAIR hyperintensities within the thrombolysis time window (44.5%) [23]. In this context, biomarkers are of interest, which are linked to tissue fate [3] and DWI intensity might serve this purpose.

It should be noted, that our analysis is an explorative and hypothesis-generating imaging study, in which only one representative slice per patient was analyzed. Based on our findings, it will be of major clinical interest to establish the relationship of DWI- intensity values and time-from-stroke-onset and tissue fate by analyzing the whole DWI lesion volume in future studies. Using such an approach, also the allocation to the 4.5 hours thrombolysis time window by DWI intensity can be investigated.

Our results rely in part on the assessment of FLAIR images as “positive” or “negative”. The raters were encouraged to look for even subtle changes by adjusting contrast and brightness and by comparing the putative lesion intensity with the intensity of the contralateral hemisphere. Following this predefined algorithm, a substantial agreement as measured by interrater kappa could be achieved. However, in this clinically relevant technique, overall agreement did not exceed 85%. This finding corroborates that the assessment of FLAIR imaging is prone to a subjective bias, especially in a clinical setting, where less rigid algorithms are applied to image rating. Despite of these findings, FLAIR imaging is an important clinical tool for the stratification of acute stroke patients and may be supported by other MRI parameters in clinical decision making in the future.

In contrast to DWI, ADC maps were not able to predict the time-dependent appearance of corresponding FLAIR lesions in acute stroke and showed no association with time-from-stroke-onset. Therefore, our results suggest that DWI intensity follows a time-dependent increase in intensity, while ADC-values do not. Several studies have described serial changes of ADC-values and DWI-intensity in acute human stroke, but focused on changes between the (hyper)acute, subacute or chronic stage. ADC values were reported to decrease in the acute stage and to increase again in the subacute and chronic stages [24]–[29]. DWI values were reported to increase between the acute and subacute stage [27] and to decrease in the chronic stage [30]. There is a substantial lack of knowledge regarding the evolution of DWI and ADC value changes *within* the acute phase of stroke. Based on these considerations, we see the need to characterize the evolution of DWI and FLAIR intensity values in humans by serial multiparametric MRI within the (hyper)acute phase of stroke.

DWI is a composite parameter of diffusion imaging and T2-imaging and thus a surrogate of very early restricted diffusion as well as following edema. ADC, on the other hand, is a quantification of diffusion alone. In our study, only DWI-intensity thresholds were able to predict the presence of FLAIR-lesions. A possible reason might be the additional information from T2 imaging present in DWI. In this respect, future studies evaluating the time-dependency of DWI from stroke-onset should shed light on the role of diffusion-weighting and T2-weighting as factors of the time-dependent intensity increase.

Importantly, we found that patient age was significantly associated with FLAIR-hyperintensities in both groups. Hence, patient age might be a confounder in the assessment of FLAIR-images for patient stratification in the acute stroke setting. This finding needs investigation to further define the predicition value of qualitative or quantitative FLAIR-imaging.

Our study has several limitations. First, a ROI based approach was chosen instead of a voxel-based analysis. A voxel-based analysis might be more accurate, but ROI-based approaches are less sensitive to spatial distortions occurring in echo planar imaging (EPI). Second, as evidenced by descriptive statistics (s. Table 1), lesion size was heterogenous in both groups, which might include a bias. Third, stroke volumes and stroke severity (based on NIHSS) were only moderate. It remains to be shown, whether our results are also applicable to patient samples with larger mean infarct volumes. Fourth, the patient groups were rather small. Our results should be validated in larger patient samples in future studies. Fifth, comparison of the results obtained at 1.5 T and 3 T is limited by a large time span between the measurements of the study groups. This led to a higher exclusion rate of images due to inferior image quality in the 1.5 T group as a results of advances in MRI techniques. This could have affected the study results. Sixth, owing to mild NIHSS values and small lesion volumes the cohort measured at 3T is not representative for patients eligible for thrombolysis. Our results must therefore be validated for a more diverse patient sample in future studies.

## Summary

In conclusion, a relative DWI-intensity threshold predicted the presence of hyperintensities in FLAIR imaging at both 1.5 and 3 T with good accuracy in a retrospective sample. Moreover, DWI-intensity values were associated with time-from-stroke-onset. These findings suggest a time-dependent increase of DWI-intensity in the hyperacute phase of stroke. Future studies should investigate the value of DWI-intensity measurement as an easily accessable estimate of lesion-age.
